# Surgical Video Understanding with Alignment-Preserving Temporal Adaptation and Action Triplet Text Alignment

**DOI:** 10.3390/bioengineering13060640

**Published:** 2026-05-29

**Authors:** Taiyo Ikeido, Ren Togo, Takahiro Ogawa, Taku Sugiyama, Saseem Poudel, Hiroyuki Sugimori, Minghui Tang, Feng Han, Hidenori Koyano, Kenji Hirata, Kohsuke Kudo, Miki Haseyama

**Affiliations:** 1 Graduate School of Information Science and Technology, Hokkaido University, Kita 14, Nishi 9, Kita-ku, Sapporo 060-0814, Hokkaido, Japan; ikeido@lmd.ist.hokudai.ac.jp; 2 Faculty of Information Science and Technology, Hokkaido University, Kita 14, Nishi 9, Kita-ku, Sapporo 060-0814, Hokkaido, Japan; togo@lmd.ist.hokudai.ac.jp (R.T.); ogawa@lmd.ist.hokudai.ac.jp (T.O.); 3 Department of Neurosurgery, Graduate School of Medicine, Hokkaido University, Kita 15, Nishi 7, Kita-ku, Sapporo 060-8638, Hokkaido, Japan; takus1113@med.hokudai.ac.jp; 4 Department of Gastroenterological Surgery II, Graduate School of Medicine, Hokkaido University, Kita 15, Nishi 7, Kita-ku, Sapporo 060-8638, Hokkaido, Japan; saseem@gmail.com; 5 Faculty of Health Sciences, Hokkaido University, Kita 12, Nishi 5, Kita-ku, Sapporo 060-0812, Hokkaido, Japan; sugimori@hs.hokudai.ac.jp; 6 Department of Diagnostic Imaging, Graduate School of Medicine, Hokkaido University, Kita 15, Nishi 7, Kita-ku, Sapporo 060-8638, Hokkaido, Japankhirata@pop.med.hokudai.ac.jp (K.H.); kkudo@med.hokudai.ac.jp (K.K.); 7 Division of AI Support for Medical Research, Faculty of Medicine, Hokkaido University, Kita 15, Nishi 7, Kita-ku, Sapporo 060-8638, Hokkaido, Japan; hanfenghokudai@gmail.com; 8 Technical Support Center, Graduate School of Medicine, Hokkaido University, Kita 15, Nishi 7, Kita-ku, Sapporo 060-8638, Hokkaido, Japan; koyano@med.hokudai.ac.jp

**Keywords:** surgical video understanding, vision–language pretraining, temporal adaptation, text prototype matching, few-shot recognition, CholecT50

## Abstract

Surgical workflow understanding requires recognizing procedural phases and fine-grained activities from long-horizon videos, yet acquiring dense annotations for surgical video analysis is costly and requires medical expertise. To address this challenge, we present a text-guided and annotation-efficient framework for surgical video understanding based on a frozen surgical vision–language-pretrained (VLP) encoder and a lightweight temporal adapter. The frozen SurgVLP image encoder provides frame-level visual embeddings, and the temporal adapter aggregates them into clip-level representations while preserving compatibility with the pretrained visual–text embedding space. We evaluate the proposed framework on CholecT50 using text-guided prototype matching for phase recognition and few-shot triplet recognition. Experiments show that temporal adaptation improves phase recognition while preserving the pretrained SurgVLP embedding space. In particular, among the evaluated methods, the proposed Text Contrastive method with rich phase prompts achieves the highest phase recognition performance, outperforming the phase-only baseline. Furthermore, the proposed framework enables classifier-free few-shot triplet recognition in the frozen text space without training a dedicated triplet classifier. These results suggest that effective surgical video understanding under limited annotation depends not only on temporal adaptation but also on preserving alignment with the pretrained text space and using semantically informative text prompts.

## 1. Introduction

As the adoption of artificial intelligence continues to expand across many domains, the medical field has also rapidly advanced through the digitalization of medical data under the framework of medical AI [1,2,3,4,5]. Medical data includes not only images but also videos, and surgical video understanding has recently attracted increasing attention as an important problem in medical video analysis [6,7,8]. Surgical video understanding has potential applications in intraoperative assistance, surgical quality assessment, and educational support for less-experienced surgeons [6]. However, learning from surgical videos remains difficult because surgical scenes often exhibit strong visual similarity across different phases and verbs, while dense annotation requires substantial medical expertise. In particular, fine-grained labels for surgical activities are costly to obtain, making annotation efficiency a central challenge in this field [9,10,11,12].

Against this background, research on surgical video understanding has primarily developed through methods specialized for individual tasks, such as surgical phase recognition, instrument detection, and action recognition [7,13]. Although these methods have shown promising performance, they generally require task-specific annotations and supervised training for each recognition target, which limits their scalability and generalization to broader surgical video understanding settings [9,14].

To address the high annotation cost, recent studies have explored self-supervised and multimodal representation learning in medical imaging from several complementary angles. Togo et al. [15] proposed an exponential dissimilarity–dispersion family for domain-specific representation learning, providing a principled way to align representations with the underlying data geometry. The same group also introduced ConcVAE [16], a conceptual variational framework designed to yield interpretable, concept-aligned representations from limited supervision. Ye et al. [17] extended this line of work to the multi-modal medical setting by proposing a continual self-supervised learning framework that incrementally integrates new modalities while preserving previously learned representations. Li et al. [18] further explored region-guided masked image modeling (RGMIM) tailored to X-ray images, demonstrating that anatomy-aware masking improves representation quality under limited annotations. These works collectively show the value of label-efficient representation learning in medical imaging. However, they primarily target static images and do not explicitly model the long-horizon temporal structure that characterizes surgical videos.

In contrast, SurgVLP [19] learns a medical-domain-specific shared embedding space by aligning visual features extracted from surgical educational videos with textual descriptions generated from automatic speech recognition (ASR) systems [20]. This vision–language pretraining framework enables text-guided recognition of semantic concepts in surgical videos, including phases, instruments, verbs, and targets, while reducing dependence on manually annotated labels.

Nevertheless, existing surgical vision–language pretraining frameworks are primarily based on frame-level alignment and do not explicitly model temporal dependencies across video clips. As a result, they do not fully exploit the sequential structure of surgical workflows. A straightforward extension would be to jointly optimize downstream temporal and fine-grained recognition objectives on top of the pretrained representation. However, our experiments reveal that naive joint training with binary classification objectives can degrade the original vision–language alignment space, which is essential for text-guided recognition. This indicates that, in surgical vision–language learning, preserving the pretrained alignment space is as important as incorporating temporal context.

In this study, we present a text-guided and annotation-efficient framework for surgical video understanding built on a frozen SurgVLP image encoder and a lightweight temporal adapter. Instead of fine-tuning the full vision–language model, we preserve the pretrained embedding space and introduce temporal reasoning only through the adapter. For phase recognition, temporally enhanced video representations are matched with text prototypes in the shared SurgVLP embedding space. Furthermore, to avoid the alignment degradation caused by naive BCE-based joint learning for triplet prediction, we introduce a Text Contrastive Loss that preserves alignment by computing the supervision directly in the shared text embedding space.

We evaluate the proposed framework on CholecT50 [21], a benchmark dataset of laparoscopic cholecystectomy with phase and triplet annotations. Our experiments show that temporal adaptation substantially improves phase recognition over the frame-level baseline, while preserving compatibility with the frozen surgical VLP embedding space. We also show that BCE-based joint learning degrades the alignment space, whereas the proposed text-space contrastive design better preserves text-guided recognition. In addition, we demonstrate that procedure-specific rich text descriptions significantly improve prototype matching without additional fine-tuning, and enable few-shot triplet recognition.

The proposed framework differs from existing surgical vision–language pretraining approaches in three key aspects. First, clip-level temporal adaptation through a lightweight adapter that preserves the pretrained embedding geometry. Second, auxiliary triplet supervision performed directly inside the shared SurgVLP text embedding space, which our experiments suggest is beneficial for retaining text-guided recognition capability. Third, classifier-free few-shot triplet recognition through nearest-neighbor retrieval against natural-language prototypes, helping to reduce the annotation burden. Together, these design choices position our work as an annotation-efficient and alignment-preserving extension of surgical vision–language pretraining, rather than as a competitor to fully supervised end-to-end pipelines.

The main contributions of this work can be summarized as follows:

- We propose an annotation-efficient surgical video understanding framework that combines a frozen surgical vision–language pretrained encoder with a lightweight temporal adapter for text-guided phase recognition.
- We identify that naive BCE-based joint learning degrades the pretrained vision–language alignment space, and introduce a Text Contrastive Loss that performs the supervision directly in the shared text embedding space.
- We demonstrate that text prompt specificity critically affects VLP-based surgical video recognition, and that the proposed framework enable few-shot triplet recognition on CholecT50.

## 2. Related Work

This section reviews prior studies related to the proposed framework from three perspectives. Section 2.1 summarizes task-specific supervised approaches for surgical video understanding, including phase recognition and fine-grained activity recognition. Section 2.2 reviews vision–language pretraining methods for surgical videos, and Section 2.3 discusses temporal adaptation and text-guided recognition, which motivate our alignment-preserving temporal adapter.

### 2.1. Surgical Video Understanding with Task-Specific Supervision

Task-specific supervised learning has been the traditional approach to surgical video understanding. In this paradigm, researchers develop separate models and label sets for individual recognition targets: phase recognition, instrument detection, verb recognition, and so forth. This section reviews the strengths and limitations of this approach, which motivates the shift toward more general-purpose methods like vision–language pretraining.

**Figure 1:**
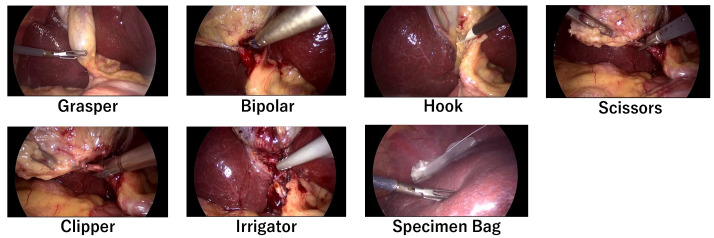
Representative frames showing different surgical instruments observed in laparoscopic cholecystectomy videos. Several frames exhibit highly similar visual appearances in terms of instruments, anatomy, and viewpoint, making frame-level discrimination difficult without temporal context.

Surgical video understanding has emerged as a central topic in surgical data science, aiming to analyze procedural workflows and fine-grained activities from recorded operations. Early studies in this field mainly focused on task-specific supervised problems such as surgical phase recognition, instrument detection, and surgical verb recognition [7,13,22,23]. Among these tasks, surgical phase recognition has been widely studied as a coarse representation of workflow progress, while fine-grained activity recognition aims to identify detailed interactions such as instrument–verb–target triplets. Datasets such as CholecT50 have enabled this line of research by providing multi-level annotations for laparoscopic cholecystectomy videos [21]. As illustrated in Figure 1, many frames in laparoscopic cholecystectomy videos exhibit highly similar visual appearances in terms of instruments, anatomy, and camera viewpoint, making frame-level discrimination difficult without temporal context.

Although these supervised approaches have achieved strong performance, they typically require task-specific annotations and dedicated training for each recognition target. This limits their scalability to broader surgical workflow understanding settings, especially when fine-grained labels must be obtained with expert involvement. Therefore, reducing annotation costs while preserving semantic recognition capability remains an important challenge in surgical video analysis.

### 2.2. Vision–Language Pretraining for Surgical Videos

**Figure 2:**
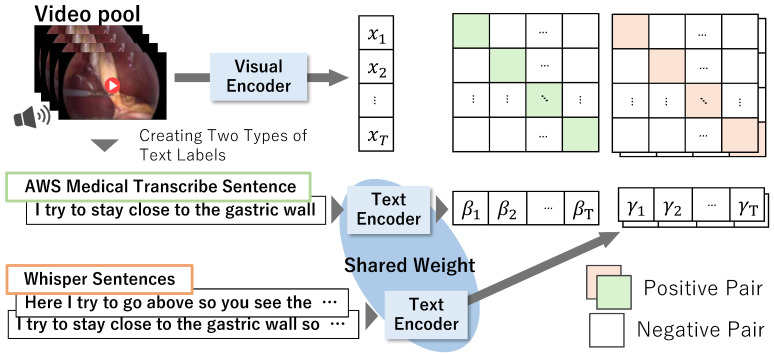
Overview of the SurgVLP framework. SurgVLP learns a shared visual–text embedding space by aligning surgical video frames with ASR-derived textual descriptions.

To alleviate the dependence on dense manual annotation, recent work has explored multimodal representation learning for surgical videos. A representative approach is SurgVLP, whose overall framework is illustrated in Figure 2. SurgVLP learns a shared visual–text embedding space by aligning surgical video frames with textual descriptions extracted from medical educational videos [19]. Inspired by vision–language pretraining methods such as CLIP [24], this framework enables text-guided recognition of semantic concepts in surgical videos, including phases, instruments, verbs, and targets, while reducing the need for task-specific labels. Text-guided retrieval has also been explored in broader multimedia settings, where text-based queries are used to retrieve semantically relevant visual content [25]. Furthermore, continual vision–language pretraining has recently been explored in the medical domain to extend pretrained representations across incremental imaging modalities [26].

This multimodal formulation is particularly attractive for surgical video understanding because it allows recognition to be performed through similarity matching against text prototypes rather than fully supervised classification alone. However, SurgVLP primarily focuses on visual–text alignment from surgical video lectures and does not explicitly study downstream temporal adaptation for preserving the pretrained alignment space during surgical workflow recognition. This limitation motivates the need for temporal modeling mechanisms that can exploit the sequential structure of surgical workflows while retaining compatibility with text-guided recognition.

Recent surgical VLP studies have further extended this direction. HecVL introduces hierarchical video–language pretraining by aligning surgical videos with clip-level, phase-level, and video-level textual descriptions, enabling zero-shot surgical phase recognition and improving transferability across procedures and institutions [27]. Its strength lies in modeling multi-level surgical semantics, but its main focus is zero-shot phase recognition rather than downstream alignment-preserving temporal adaptation for fine-grained triplet understanding. PeskaVLP further addresses the limitations of noisy and incomplete surgical narrations by introducing procedure-aware knowledge augmentation with LLM-refined textual supervision, hard negative construction, and DTW-based temporal alignment [28]. Its strength is the improvement of surgical VLP pretraining itself through richer procedural supervision; however, it does not specifically investigate how a frozen surgical VLP encoder can be adapted to downstream temporal recognition while preserving its original visual–text embedding geometry. In contrast, our work keeps the pretrained SurgVLP encoder frozen and studies lightweight temporal adaptation and text-space triplet supervision for annotation-efficient phase and triplet recognition.

### 2.3. Temporal Adaptation and Text-Guided Recognition

**Figure 3:**
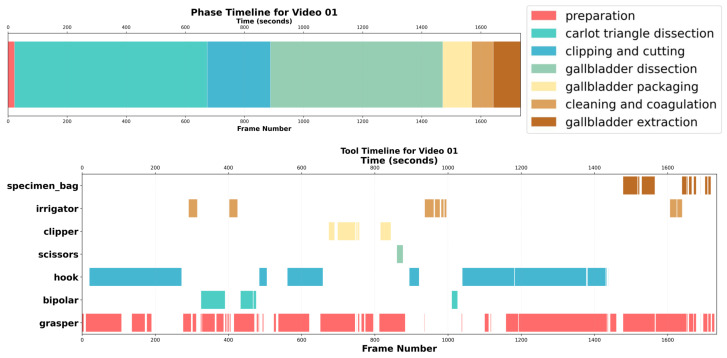
Example temporal annotation profile of a surgical video. The top row shows the phase timeline, and the bottom row shows frame-wise instrument occurrences. Surgical phases form relatively long contiguous segments, whereas instrument appearances are sparse and intermittent, highlighting the importance of temporal modeling for surgical video understanding.

**Figure A1:**
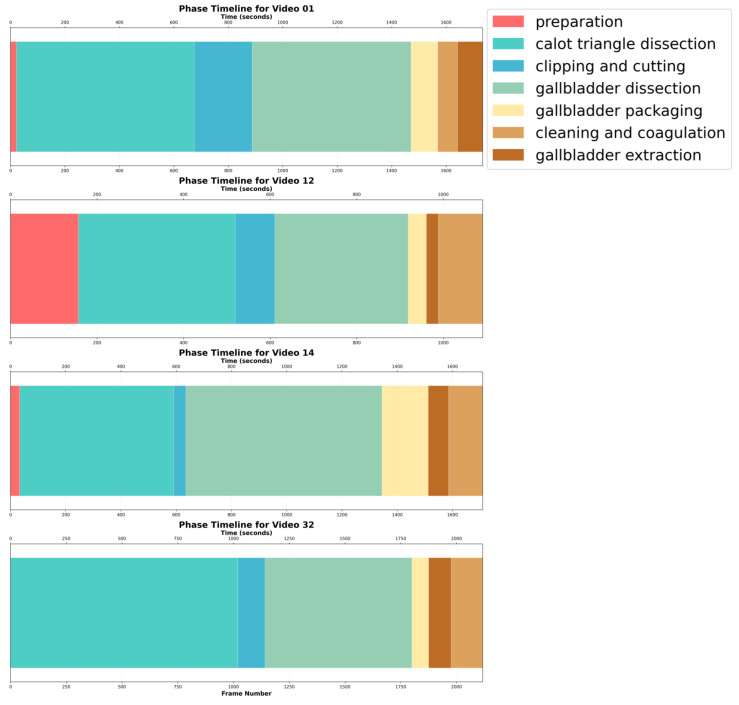
Examples of ground-truth phase timelines showing inter-video variation in phase duration and transitions.

**Figure A2:**
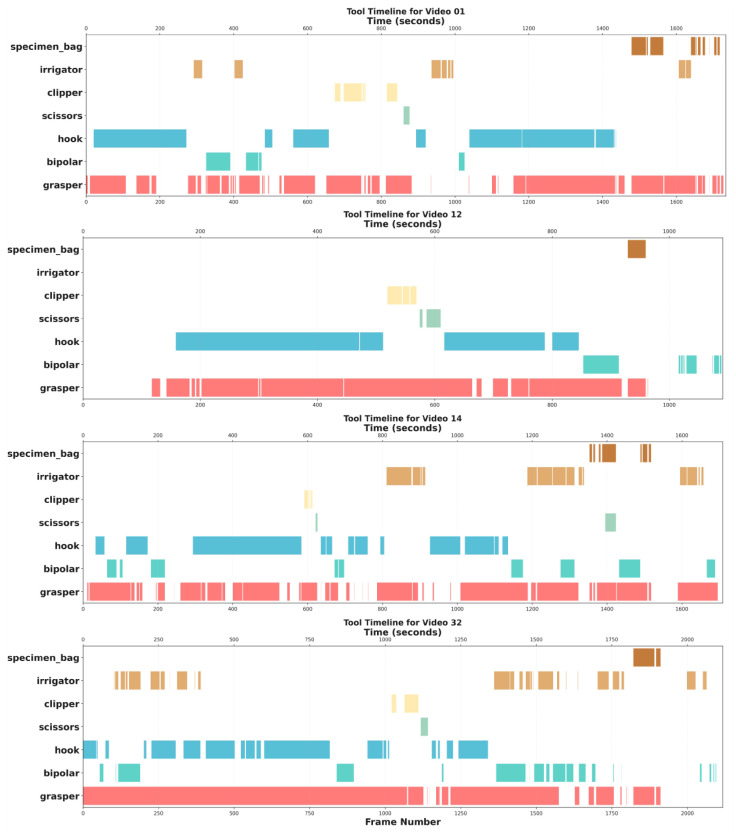
Examples of ground-truth instrument timelines showing sparse and variable instrument occurrence across videos.

Temporal modeling is essential in video understanding because surgical procedures unfold as ordered sequences of phases and verbs. As illustrated in Figure 3, within a single surgical video, phases tend to form relatively long contiguous segments, whereas instrument occurrences are sparse and intermittent over time. Moreover, this temporal structure is not fixed across cases: the duration and transition patterns of phases vary across videos (Figure A1), and the occurrence patterns of instruments are also highly variable from one procedure to another (Figure A2). These observations highlight that surgical video understanding requires not only frame-level semantic recognition but also temporal modeling that can capture both intra-video temporal continuity and inter-video variation.

Prior video understanding studies have introduced recurrent networks, temporal convolutions, and Transformer-based architectures to capture temporal dependencies, and temporal pretraining methods such as TIME have further shown the value of self-attention-based temporal reasoning in general video analysis [29]. In the medical domain, temporally aware adaptation has also been explored for longitudinal clinical data understanding [30]. These developments suggest that temporal adaptation is a promising direction for extending frame-based surgical VLP representations to clip-level recognition.

At the same time, text-guided recognition introduces a challenge that is less emphasized in conventional supervised video classification: preserving the pretrained vision–language alignment space during downstream adaptation. Contrastive learning has become a foundational technique in medical AI for learning discriminative representations from limited annotations [31]. Our experimental findings show that, although temporal adaptation improves phase recognition over frame-level prototype matching, naive BCE-based joint learning for fine-grained triplet prediction can degrade the original alignment space. This motivates a different design principle from standard joint classification pipelines: rather than replacing the text-guided embedding space with task-specific classification heads, downstream training should preserve compatibility with the pretrained text space. Our work is positioned at this intersection by combining a frozen surgical VLP encoder, a lightweight temporal adapter, and text-space alignment for phase recognition and few-shot triplet understanding.

## 3. Methods

This section describes the proposed text-guided framework for surgical video understanding. The framework realizes the three contributions stated in Section 1 as follows. The first contribution, an annotation-efficient framework that couples a frozen SurgVLP encoder with a lightweight temporal adapter, is presented in Section 3.1 and Section 3.2. The second contribution, the Text Contrastive Loss that preserves the pretrained vision–language alignment space, is introduced in Section 3.4. The third contribution, the use of procedure-aware rich text prompts that enable few-shot triplet recognition, is integrated into Section 3.3 and Section 3.4. The overall training objective that combines these components is given in Section 3.5. Trainable parameters and the computational footprint of the proposed framework are summarized in Section 3.6.

We first extract frame-level visual embeddings using the frozen image encoder of SurgVLP in order to preserve the original visual–text alignment space (Section 3.1). To incorporate temporal context, we introduce a lightweight Transformer-based temporal adapter that aggregates frame features into a clip-level representation while maintaining compatibility with the pretrained embedding geometry (Section 3.2).

**Figure 4:**
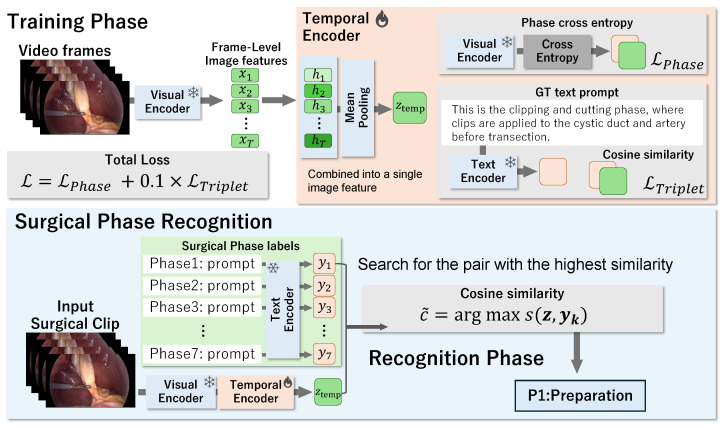
Overview of the proposed framework: Integrating temporal dynamics and triplet information for few-shot surgical task recognition.

During training, phase supervision is applied through a phase classification head (Section 3.3), while fine-grained triplet supervision is introduced through text-based alignment to SurgVLP triplet embeddings constructed from rich phase-aware natural-language descriptions (Section 3.4). At evaluation time, we assess the learned clip representation in a text-guided manner by matching it against phase text prototypes encoded by the frozen SurgVLP text encoder. An overview of the proposed framework, including temporal adaptation and text-guided triplet alignment, is shown in Figure 4.

### 3.1. Frame-Level Visual Feature Extraction

We represent an input surgical clip consisting of *T* sampled frames as

(1) $$\{I_{1},I_{2},\ldots ,I_{T}\}.$$

Each frame $I_{t}$ is resized to 448×448 pixels following the input resolution adopted in the K600-pretrained TimeSformer checkpoint [29], and is then independently encoded by the image encoder $f_{\theta }$ of SurgVLP to obtain a *D*-dimensional visual embedding:

(2) $$x_{t}=f_{\theta }\left(I_{t}\right),\;x_{t}\in R^{D}.$$

Here, $f_{\theta }$ is a CLIP-based vision–language image encoder pretrained on large-scale surgical educational videos [19], and the embedding dimension is D=512 following the original SurgVLP configuration. In our framework, this encoder is kept frozen throughout training in order to preserve the pretrained visual–text embedding space. This design allows the model to retain compatibility with text representations derived from SurgVLP prompts while adapting only a lightweight temporal module for downstream recognition.

### 3.2. Temporal Adapter and Clip-Level Aggregation

To incorporate temporal context into the frame-level SurgVLP features, we introduce a Transformer-based temporal adapter $g_{\phi }$ that models interactions among frame embeddings while preserving the original feature dimensionality. The adapter receives the frame sequence

(3) $$X=\left[x_{1},x_{2},\ldots ,x_{T}\right]\in R^{T\times D}$$

and produces temporally contextualized features

(4) $$H=g_{\phi }\left(X\right)\in R^{T\times D}.$$

Because the temporal adapter preserves the embedding dimensionality of SurgVLP, the resulting representations remain compatible with the pretrained visual–text embedding space. This property is important in our framework because downstream evaluation is performed through similarity to text prototypes rather than only through task-specific classification heads.

The clip-level representation $z\in R^{D}$ is obtained by mean pooling over the temporal dimension:

(5) $$z=\frac{1}{T}\sum_{t=1}^{T}H_{t}.$$

In the experiments, we also compare this with a TIME-initialized temporal encoder [29] as an alternative temporal adapter, as well as different aggregation strategies including CLS-token aggregation and weighted pooling, to examine how temporal modeling and aggregation affect text-guided recognition.

#### Architecture Details

The temporal adapter $g_{\phi }$ is implemented as a stack of $N_{L}=2$ Transformer encoder layers operating on the frozen SurgVLP frame embedding sequence of dimension D=512. Each layer consists of multi-head self-attention with h=8 heads, followed by a position-wise feed-forward network with hidden dimension $d_{\mathrm{ff}}=2048$. The adapter preserves the input dimension *D* throughout, ensuring compatibility with the pretrained SurgVLP text space. The architectural hyperparameters are summarized in Table 1.

We deliberately adopt a lightweight adapter instead of full end-to-end fine-tuning because our goal is not to relearn the entire visual representation, but to inject temporal context while retaining compatibility with the pretrained text space. Mean pooling is used as the default clip aggregation strategy because it provides a stable global summary of the temporally contextualized frame features without introducing an additional trainable token or attention module. This setting is also consistent with our objective of minimizing architectural changes around the frozen SurgVLP representation.

### 3.3. Phase Training and Text-Guided Phase Evaluation

Given the clip-level visual representation $z\in R^{D}$, phase supervision is applied during training through a trainable phase classification head. Specifically, we use a linear classifier $h_{\mathrm{phase}}:R^{D}\to R^{K}$ to predict logits over *K* surgical phase categories:

(6) $$o=h_{\mathrm{phase}}\left(z\right)\in R^{K},$$

where K=7 denotes the number of phase classes.

The phase prediction probability for class *k* is obtained by softmax:

(7) $$p_{k}=\frac{\exp(o_{k})}{\sum_{m=1}^{K}\exp\left(o_{m}\right)}.$$

Let *c* denote the ground-truth phase label. The phase loss is defined as the cross-entropy loss:

(8) $$L_{\mathrm{phase}}=-\log p_{c}.$$

Although phase supervision is applied through a classifier during training, we evaluate phase recognition in a text-guided manner in order to measure compatibility with the pretrained SurgVLP embedding space. Following the few-shot evaluation protocols established in vision–language models [19,24], each candidate surgical phase is converted into a textual prompt and encoded by the frozen SurgVLP text encoder to obtain a phase prototype $y_{k}\in R^{D}$. The similarity between the clip representation and a phase text prototype is computed using cosine similarity:

(9) $$s\left(z,y_{k}\right)=\frac{z^{⊤}y_{k}}{{∥z∥}_{2}{∥y_{k}∥}_{2}}.$$

The predicted phase is then determined as

(10) $$\hat{c}=\arg\max_{k}s\left(z,y_{k}\right).$$

This evaluation protocol allows us to directly assess whether temporal adaptation and triplet supervision preserve compatibility with the pretrained visual–text embedding space. It also enables systematic analysis of prompt design by varying the phase text templates while keeping the visual encoder fixed.

### 3.4. Triplet Supervision in the SurgVLP Text Space

To incorporate fine-grained procedural semantics, we introduce triplet-level supervision on top of the clip representation z. In the dataset, each fine-grained activity is represented as a triplet consisting of instrument, verb, and target. A straightforward baseline is to treat triplet prediction as a 100-class multi-label classification problem and optimize a binary cross-entropy (BCE) loss with a trainable triplet head. However, such supervision does not explicitly preserve compatibility with the pretrained SurgVLP text space.

To preserve text-space compatibility, we instead use a text-based triplet supervision strategy. For each candidate triplet (i,v,r) occurring during phase *p*, we construct a rich natural-language sentence by concatenating a triplet action description with a phase-specific contextual description, as summarized in Table 2:

(11) $$\begin{array}{cc} s_{t,p} & ="\mathrm{This}\mathrm{surgical}\mathrm{clip}\mathrm{uses}\left\{i\right\}\mathrm{to}\left\{v\right\}\mathrm{the}\left\{r\right\}.\left\{\rho_{p}\right\}", \end{array}$$

where $\rho_{p}$ is the rich phase description for phase *p* (e.g., *“This is the gallbladder dissection phase, where the gallbladder is detached from the liver bed using electrosurgery."*). This template shares the same phase description vocabulary used in text-guided phase evaluation, thereby encouraging the adapted visual representation to remain consistent with the pretrained visual–text embedding space.

Each such sentence is encoded by the frozen SurgVLP text encoder to obtain a normalized triplet text embedding $q_{t,p}\in R^{D}$. In practice, we precompute embeddings for all 100×7=700 triplet–phase sentences and keep them fixed during training.

Given an input clip, let $\left(t^{*},p^{*}\right)$ denote the dominant triplet and phase pair assigned to that clip. In practice, a clip may contain multiple frame-level triplet annotations. To obtain a single auxiliary supervision target, we assign each clip the dominant triplet–phase pair, defined as the pair that appears most frequently within the temporal extent of the clip. Here, frequency is counted over the annotated frames belonging to the clip. If multiple pairs have the same maximum frequency, we select the pair appearing at the temporal center of the clip; if the tie still remains, we choose the earliest occurring pair. This dominant-pair assignment provides a deterministic mapping from frame-level multi-label annotations to a single clip-level text alignment target. We retrieve the corresponding text prototype $q_{t^{*},p^{*}}$ and supervise the clip representation by cosine alignment:

(12) $$L_{\mathrm{triplet}}=1-\frac{z^{⊤}q_{t^{*},p^{*}}}{{∥z∥}_{2}{∥q_{t^{*},p^{*}}∥}_{2}}.$$

This objective encourages the temporally adapted visual representation to remain close to the SurgVLP text embedding of the corresponding fine-grained procedural description. Crucially, by using the same rich phase descriptions as the phase evaluation prototypes, the triplet supervision reinforces the phase-discriminative structure of the embedding space. Unlike BCE-based classification, the proposed formulation performs triplet supervision directly in the pretrained text space, which is more compatible with the text-guided evaluation protocol used for phase recognition and few-shot triplet inference.

### 3.5. Training Objective

The final training objective for the proposed framework combines the phase classification loss and the fine-grained triplet supervision loss:

(13) $$L=L_{\mathrm{phase}}+\lambda_{\mathrm{triplet}}L_{\mathrm{triplet}}.$$

where $L_{\mathrm{phase}}$ is the phase cross-entropy loss and $L_{\mathrm{triplet}}$ is the proposed text-space triplet alignment loss or the BCE-based triplet loss used as a baseline. The hyperparameter $\lambda_{\mathrm{triplet}}$ controls the weight of the fine-grained triplet supervision.

#### Selection of $\lambda_{\mathrm{triplet}}$

The weight $\lambda_{\mathrm{triplet}}$ is selected based on validation-set phase recognition accuracy. We evaluated values in the range $\lambda_{\mathrm{triplet}}\in \left\{0.1,0.5,1.0\right\}$ and observed that $\lambda_{\mathrm{triplet}}=0.1$ provided the best balance between phase-discriminative learning and fine-grained triplet alignment. Increasing $\lambda_{\mathrm{triplet}}$ beyond this value caused the adapted embedding to drift away from the phase-discriminative region of the shared text space (cf. Section 4.3). Accordingly, we use $\lambda_{\mathrm{triplet}}=0.1$ as the default in all subsequent experiments and report the sensitivity to this hyperparameter in the ablation study. As a practical guideline, our analysis suggests that $\lambda_{\mathrm{triplet}}$ in the range [0.05,0.2] provides a good trade-off between the two objectives under the proposed text-space formulation.

### 3.6. Trainable Parameters and Computational Footprint

A central design principle of the proposed framework is annotation efficiency through parameter efficiency: the pretrained SurgVLP image and text encoders are kept frozen, and only a small temporal module and a lightweight phase head are optimized. Table 3 summarizes the parameter count of each component.

Only the temporal adapter and the linear phase head are updated during training. The temporal adapter contains approximately 11 million trainable parameters, while the phase head adds a negligible D·K+K=3591 parameters. This parameter-efficient design not only reduces training cost but also helps preserve the pretrained SurgVLP embedding space, which is critical for the text-guided recognition protocol used in our evaluation.

#### Computational Footprint

All experiments are conducted on a workstation equipped with an NVIDIA RTX 6000 Ada Generation GPU (48 GB VRAM, CUDA 12.2, driver version 535, Nvidia, Santa Clara, CA, USA) and an Intel Core i7-14700K CPU (20 cores, 28 threads, Intel, Santa Clara, CA, USA) with 64 GB of system memory, running Ubuntu 24.04 LTS. The framework is implemented in PyTorch v2.9.1 with CUDA 12.8 (PyTorch Foundation, San Francisco, CA, USA). Because the SurgVLP image encoder is frozen, its frame-level embeddings can be precomputed once and cached, so that clip-level inference only requires forward passes through the lightweight temporal adapter, the linear phase head, and the cosine-similarity matching against the precomputed text prototypes. Under this protocol, processing a single 20-frame clip on the above hardware takes only a few milliseconds at the adapter and head stage, which is well below the 1 Hz annotation rate of CholecT50 and clearly compatible with real-time clip-level usage at typical surgical-video frame rates. Reported training runs converge within the early-stopping budget of 20 epochs (see Section 4.1), confirming that the proposed framework remains computationally lightweight despite leveraging a large-scale frozen vision–language backbone.

## 4. Experiments

This section describes the experimental setup used to evaluate the proposed text-guided and annotation-efficient framework for surgical video understanding. We conduct all experiments on CholecT50 and evaluate the learned clip representations under a text-guided prototype matching protocol for phase recognition and few-shot triplet recognition.

### 4.1. Experimental Setting

We evaluate the proposed framework on CholecT50 [21], a benchmark dataset of laparoscopic cholecystectomy videos consisting of 50 videos annotated at 1 fps with surgical phase and instrument–verb–target triplet labels. Following the standard split, 40 videos are used for training, 5 for validation, and 5 for testing. Each video is segmented into non-overlapping clips of 20 frames.

Frame-level visual features are extracted by the frozen SurgVLP image encoder and fed into a lightweight Transformer temporal adapter, whose outputs are mean-pooled into a single clip-level embedding $z\in R^{D}$. The SurgVLP image and text encoders are kept frozen throughout training, and only the temporal adapter and the phase classification head are optimized. We train the model for up to 20 epochs with early stopping (patience = 5) using AdamW (learning rates of $10^{-5}$ for the temporal adapter and $10^{-4}$ for the phase head, batch size =16). The default triplet loss weight is set to $\lambda_{\mathrm{triplet}}=0.1$ based on validation accuracy.

Our experimental design follows the principle of minimizing changes to the pretrained SurgVLP representation. Accordingly, the image encoder and text encoder are kept frozen in all settings, and adaptation is restricted to the temporal module and the phase supervision head. This setting allows us to isolate the effect of temporal adaptation and auxiliary triplet text alignment without conflating it with full multimodal fine-tuning. It also makes the comparison between the CE-only baseline, the BCE-based triplet baseline, and the proposed text-space alignment strategy more interpretable from the viewpoint of compatibility with the frozen text space.

For **phase recognition**, we adopt a text-guided prototype matching protocol. A text prototype for each of the seven surgical phases is obtained by encoding a phase description with the frozen SurgVLP text encoder. The predicted phase is the prototype with the highest cosine similarity to z, and we report test accuracy. We compare two prompt designs: a *plain* template containing only the phase name and a *rich* template with procedure-specific descriptive context. The full rich prompt definitions are provided in Appendix A.

For **few-shot triplet recognition**, we explicitly distinguish training-time supervision from test-time evaluation. During training, the Text Contrastive model aligns the clip embedding to triplet–phase text descriptions in the SurgVLP text space. At test time, however, each of the 100 candidate triplets is converted into a simpler natural-language sentence of the form *“This surgical clip uses {instrument} to {verb} the {target}.”* and embedded by the frozen SurgVLP text encoder. The predicted triplet is obtained by nearest-neighbor retrieval in the text embedding space, without training a dedicated triplet classifier for inference. We report accuracy for the full triplet as well as for each component (instrument, verb, and target).

All experiments are conducted on a workstation equipped with an NVIDIA RTX 6000 Ada Generation GPU (48 GB VRAM), an Intel Core i7-14700K CPU, and 64 GB of system memory, running Ubuntu 24.04 LTS with PyTorch and CUDA 12.2 (see Section 3.6 for details on the computational footprint).

### 4.2. Main Results

#### 4.2.1. Phase Recognition

Table 4 summarizes phase recognition accuracy on CholecT50. Without fine-tuning, SurgVLP features achieve 0.265 with plain prototypes, confirming that the pretrained text–image alignment already captures coarse phase semantics. Training the temporal adapter with phase supervision only improves accuracy to 0.368 with rich prototypes but slightly degrades performance under plain prototypes (0.297), suggesting that CE-only training shifts the adapted embedding away from the pretrained text space. In contrast, the Text Contrastive model achieves the best result of **0.518**. These results indicate that temporal adaptation is most effective when the learned clip embedding remains compatible with a semantically informative text space.

#### 4.2.2. Few-Shot Triplet Recognition

Table 5 reports few-shot triplet recognition accuracy evaluated with the natural-language retrieval template. Phase-only training substantially degrades triplet accuracy compared with no fine-tuning (0.013 vs. 0.071), indicating that phase CE supervision alone does not preserve the pretrained text–image alignment required for text-based retrieval. In contrast, the Text Contrastive model improves triplet accuracy to **0.165**, instrument accuracy to **0.505**, and target accuracy to **0.558**. This result suggests that aligning clip embeddings to triplet–phase text descriptions during training implicitly encodes fine-grained procedural information that transfers to retrieval-based triplet recognition. Verb accuracy remains relatively low across all settings, implying that action semantics are still difficult to capture with prototype matching in the current frozen text space.

This suggests that the proposed text-space alignment mainly reinforces appearance-associated semantics, such as instruments and anatomical objects, which are more directly reflected in the frozen visual representation. By contrast, surgical verbs often depend on subtle motion patterns and interaction dynamics distributed over time, making them more difficult to recover through a single clip-level embedding and nearest-neighbor retrieval in the frozen text space.

These findings indicate that the current framework is effective for transferring structured semantic cues from text to video, but that stronger motion-sensitive adaptation may be necessary for more reliable verb-level understanding.

#### 4.2.3. Comparison with Existing Surgical VLP Models

To position our framework relative to existing surgical vision–language pretraining methods, Table 6 compares the proposed method with three representative surgical VLP models—SurgVLP [19], HecVL [27], and PeskaVLP [28]—all evaluated under the same zero-/few-shot prototype matching protocol on the CholecT50 test split. We deliberately scope this comparison to surgical VLP models with matching annotation budgets, because the proposed framework is designed as an annotation-efficient and alignment-preserving extension of surgical vision–language pretraining rather than as a direct competitor to fully supervised end-to-end pipelines.

Among the surgical VLP baselines, HecVL achieves 0.371, SurgVLP achieves 0.334, and PeskaVLP achieves 0.302 in overall phase recognition accuracy. The proposed Text Contrastive framework reaches 0.518 with rich prototypes, substantially outperforming all three surgical VLP baselines under the same evaluation regime. This gain confirms that the proposed combination of lightweight temporal adaptation and text-space alignment yields a stronger pretrained-text-space predictor than the current generation of surgical VLP models.

### 4.3. Ablation and Analysis

Table 7 summarizes the main ablation results for phase recognition. First, richer phase prompts substantially improve prototype matching, especially under Text Contrastive training, where the gap between plain and rich prompts reaches 0.224. Second, increasing $\lambda_{\mathrm{triplet}}$ from 0.1 to 0.5 reduces phase accuracy from 0.518 to 0.449, indicating that overly strong triplet supervision can pull the embedding away from the phase-discriminative region of the shared text space. Third, the Transformer temporal adapter outperforms the TIME-based alternative in our setting.

#### Compatibility with the Pretrained Text Space

We use text-guided prototype matching accuracy as an operational measure of compatibility with the pretrained visual–text embedding space. Since inference is performed by directly comparing the adapted clip representation with frozen SurgVLP text prototypes, successful prototype matching indicates that the adapted representation remains usable in the pretrained text space. The improvement from the no-fine-tuning baseline to the proposed Text Contrastive model suggests that temporal adaptation improves recognition while preserving practical compatibility with frozen text prototypes. The ablation on λ triplet further shows that overly strong triplet supervision degrades this compatibility, supporting the need for moderate text-space alignment.

## 5. Discussion and Limitations

The experimental results demonstrate that the proposed text-guided temporal adaptation framework can improve surgical phase recognition while partially preserving compatibility with the pretrained SurgVLP text space. In particular, Text Contrastive training achieves the best phase recognition accuracy of 0.518 with rich prompts and also improves few-shot triplet recognition to 0.165. These findings suggest that aligning clip embeddings with semantically structured text descriptions is an effective way to transfer pretrained vision–language representations to surgical video understanding.

However, several limitations remain. First, the current evaluation is conducted only on CholecT50, which consists of 50 laparoscopic cholecystectomy videos. Although this dataset is a standard benchmark, it covers a single surgical procedure and a limited number of videos. Therefore, the generalizability of the proposed framework to other procedures, institutions, or recording conditions remains unclear.

Although laparoscopic cholecystectomy is relatively standardized, other surgical procedures involve different workflow structures, anatomical contexts, and instrument sets. Thus, cross-dataset evaluation on datasets such as Cholec80 and nephrectomy or prostatectomy corpora is needed to assess generalizability beyond the current domain. In addition, institutional differences in imaging protocols, camera viewpoints, compression artifacts, and surgical conventions may affect the pretrained SurgVLP embedding space. Evaluating robustness across diverse procedures and recording conditions remains an important direction for future work. In addition, the current study does not report confidence intervals or statistical significance tests for the experimental results. Since our evaluation is based on a limited fixed test split of CholecT50, we avoid making strong statistical significance claims from the present results. Future work will evaluate the proposed framework using repeated runs, larger test sets, and cross-dataset validation, together with confidence intervals or statistical significance tests, to more rigorously assess the reliability of the observed performance differences.

Second, the results are sensitive to the design of the text prototypes used for evaluation. The large gap between plain and rich prompts indicates that recognition performance depends not only on the learned video representation but also on how explicitly the text side encodes procedural context. This implies that under-specified text templates may underestimate the true capability of the adapted representation, while highly descriptive prompts may introduce an additional source of task-specific engineering.

Third, few-shot triplet recognition remains limited, especially for verb prediction. While instrument and target accuracies improve substantially under Text Contrastive training, verb accuracy remains relatively low. This suggests that the current framework captures appearance-oriented semantics more reliably than fine-grained verb semantics. Because many surgical verbs are defined by subtle motion differences distributed over time, a clip-level prototype matching scheme in a frozen text space may be insufficient to represent them robustly.

Another limitation is the mismatch between training-time and test-time text formulations. In the current implementation, triplet supervision during training uses triplet–phase descriptions that explicitly include the surgical phase, whereas inference for few-shot triplet recognition uses simpler triplet-only natural-language templates. Although this design allows a clean retrieval-based evaluation, it also introduces a formulation gap between supervision and inference. Reducing this gap may further improve the consistency and interpretability of the learned representation.

Finally, the present study deliberately keeps the SurgVLP image and text encoders frozen in order to preserve the pretrained multimodal space and reduce training cost. While this design is attractive from the viewpoint of annotation efficiency and stability, it may also limit the degree of surgical specialization that can be achieved. Future work should investigate cross-dataset evaluation, action-sensitive temporal modeling, and adaptive text prototype design, as well as semi-frozen or parameter-efficient fine-tuning strategies for better surgical domain adaptation [15,17,26,30].

### Annotation Efficiency vs. Supervised Accuracy

The proposed framework is designed as an annotation-efficient alternative to fully supervised methods. While approaches such as EndoNet [32], TeCNO [33], and Trans-SVNet [34] achieve high accuracy with dense annotations on Cholec80 or fully annotated CholecT50 videos, our framework reduces annotation requirements by using phase labels for temporal adaptation and text-space alignment for triplet supervision. This design may trade off some absolute supervised accuracy, but it enables faster adaptation to new procedures, institutions, and recording conditions where expert annotation is costly.

## 6. Conclusions

In this study, we presented a text-guided and annotation-efficient framework for surgical video understanding based on a frozen SurgVLP encoder and a lightweight temporal adapter. By introducing temporal modeling while maintaining compatibility with the pretrained vision–language embedding space, the proposed framework enables clip-level recognition through text prototype matching.

Experiments on CholecT50 yielded three main findings. First, temporal adaptation improved phase recognition without fine-tuning the pretrained visual encoder. Second, preserving compatibility with the pretrained text-aligned space was important: naive BCE-based joint learning degraded phase recognition, whereas the proposed Text Contrastive Loss provided a more effective way to incorporate fine-grained triplet supervision. Third, procedure-aware rich text prompts substantially improved prototype-based recognition and also supported few-shot triplet recognition without a dedicated triplet classifier.

Overall, these results suggest that effective surgical video understanding under limited annotation depends not only on temporal adaptation itself but also on maintaining alignment with the pretrained text space. At the same time, several directions remain for further investigation. First, cross-dataset evaluation on other surgical datasets, such as Cholec80 and procedure-specific nephrectomy or prostatectomy corpora, is necessary to assess generalization beyond laparoscopic cholecystectomy and to examine the influence of institutional and procedural variations. Second, developing more motion-sensitive temporal models will be important for recognizing fine-grained surgical verb semantics, which may require additional motion cues such as optical flow, pose information, or kinematic features. In addition, integrating multimodal surgical data represents an important future direction. Beyond RGB surgical videos, additional modalities such as surgical audio, tool usage logs, robotic kinematics, preoperative imaging, and operative reports may provide complementary information for understanding procedural context and fine-grained surgical actions. Large-scale self-supervised or multimodal pretraining on such heterogeneous surgical data could further improve the robustness and transferability of surgical video representations, especially when dense expert annotations are limited. Third, adaptive text prototype design based on surgical ontologies, video descriptions, or expert knowledge may reduce manual prompt engineering and improve semantic coverage. Fourth, semi-frozen or parameter-efficient fine-tuning of the visual and text encoders should be explored to achieve controlled domain adaptation while preserving annotation efficiency. In addition, systematic comparison with parameter-efficient supervised learning approaches would help clarify the trade-off between annotation cost and recognition accuracy.

Finally, although the present results provide empirical evidence for the importance of preserving text-space compatibility through text-guided evaluation, more direct quantitative analyses are needed. Future work will therefore examine cosine similarity and alignment margins between adapted clip embeddings and phase text prototypes, together with embedding-space visualizations such as t-SNE, to better characterize how temporal adaptation affects the pretrained visual–text geometry. These future directions will help advance surgical video understanding toward systems that are annotation-efficient, generalizable across procedures and institutions, and capable of capturing both appearance-based and motion-based surgical semantics.

## Figures and Tables

**Table 1:** Architectural hyperparameters of the proposed Transformer-based temporal adapter $g_{\phi }$.

Component	Configuration
Number of layers ($N_{L}$)	2
Embedding dimension (*D*)	512
Number of attention heads (*h*)	8
Feed-forward hidden dimension ($d_{\mathrm{ff}}$)	2048
Positional encoding	Sinusoidal
Default clip aggregation	Mean pooling over *T*
Input embedding dimension preserved	Yes (D=512)
Input sequence length (*T*)	20 frames per clip

**Table 2:** Rich triplet–phase text template used for text-space supervision during training. Each sentence combines a triplet action description with a phase-specific rich description, matching the prototype text used in text-guided phase evaluation.

Usage	Template/Example
Template	This surgical clip uses {instrument} to {verb} the {target}. {Rich phase description for {phase}}
Example 1	This surgical clip uses grasper to dissect the cystic plate. This is the calot triangle dissection phase, where the surgeon carefully dissects the hepatocystic triangle to identify the cystic duct and artery.
Example 2	This surgical clip uses clipper to clip the cystic duct. This is the clipping and cutting phase, where clips are applied to the cystic duct and artery before transection.

**Table 3:** Parameter count of each component in the proposed framework. The SurgVLP image and text encoders remain frozen throughout training; only the temporal adapter and the phase head are optimized.

Component	Role	Trainable
SurgVLP image encoder ($f_{\theta }$)	Frame-level feature extractor	Frozen
SurgVLP text encoder	Phase/triplet prototype encoder	Frozen
Temporal adapter ($g_{\phi }$)	Clip-level temporal aggregation	∼11 M params
Phase head ($h_{\mathrm{phase}}$)	Linear classifier over K=7 phases	D·K+K=3591 params
**Total trainable parameters**		∼11 M

**Table 4:** Phase recognition accuracy on CholecT50. Plain and rich refer to the prototype template used at evaluation.

Method	Plain	Rich
No fine-tuning	0.265	0.319
Phase-only (CE)	0.297	0.368
Text Contrastive (λ=0.1)	0.294	**0.518**

**Table 5:** Few-shot triplet recognition accuracy on CholecT50 using natural-language retrieval templates. Inference is performed by nearest-neighbor matching in the frozen text space rather than by a dedicated triplet classifier.

Method	Triplet	Instrument	Verb	Target
No fine-tuning	0.071	0.322	0.340	0.381
Phase-only (CE)	0.013	0.157	0.249	0.190
Text Contrastive (λ=0.1)	**0.165**	**0.505**	0.338	**0.558**

**Table 6:** Comparison with existing surgical vision–language pretraining models on CholecT50 under a zero-/few-shot prototype matching protocol. All baselines use no phase-specific labels during pretraining, while the proposed framework uses phase labels only to train a lightweight temporal adapter and a linear phase head on top of a frozen SurgVLP encoder.

Method	Supervision Regime	Phase Acc.
*Zero-shot prototype matching (no phase labels)*		
HecVL [27]	Vision–language pretraining only	0.371
SurgVLP [19]	Vision–language pretraining only	0.334
PeskaVLP [28]	Vision–language pretraining only	0.302
*Few-shot/annotation-efficient (proposed framework, frozen SurgVLP encoder)*		
Phase-only (CE)	Phase labels, frozen encoder	0.368
**Text Contrastive (ours)**	Phase + text-space triplet, frozen encoder	0.518

**Table 7:** Summary of ablation results for phase recognition on CholecT50.

Setting	Prompt	$\lambda_{\mathrm{triplet}}$	Accuracy
Phase-only (CE, Transformer)	Rich	—	0.368
Text Contrastive (Transformer)	Plain	0.1	0.294
Text Contrastive (Transformer)	Rich	0.1	**0.518**
Text Contrastive (Transformer)	Rich	0.5	0.449
Text Contrastive (TIME)	Rich	0.1	0.273

## Data Availability

The datasets presented in this article are not readily available because they were obtained from a third-party dataset provider, and access is subject to the provider’s terms and conditions. Requests to access the datasets should be directed to the official CholecT50 dataset repository maintained by CAMMA.

## Appendix A. Phase Prompt Definitions

Table A1 lists the full rich phase prompts used for text-guided prototype matching.

**Table A1:** Rich phase text prompts used for text-guided prototype matching.

Phase	Rich Prompt
Preparation	This is the preparation phase of laparoscopic cholecystectomy, where the surgeon positions trocars and inspects the abdominal cavity.
Calot Triangle Dissection	This is the Calot triangle dissection phase, where the surgeon carefully dissects the hepatocystic triangle to identify the cystic duct and cystic artery.
Clipping and Cutting	This is the clipping and cutting phase, where clips are applied to the cystic duct and cystic artery before transection.
Gallbladder Dissection	This is the gallbladder dissection phase, where the gallbladder is detached from the liver bed using electrosurgery.
Gallbladder Packaging	This is the gallbladder packaging phase, where the dissected gallbladder is placed into a retrieval bag.
Cleaning and Coagulation	This is the cleaning and coagulation phase, where the surgical field is irrigated and bleeding is controlled.
Gallbladder Extraction	This is the gallbladder extraction phase, where the bagged gallbladder is removed through the trocar port.
